# Cone Beam Computed Tomography of Bonejaws in Patients With Primary Osteoporosis: A Systematic Review

**DOI:** 10.1007/s11914-026-00972-3

**Published:** 2026-05-21

**Authors:** Irene Frassineti, Lorenzo Cinci, Andrea Magnini, Maria Pisano, Luigi Barbato, Francesco Cairo, Luisella Cianferotti, Cosimo Nardi

**Affiliations:** 1 Department of Experimental and Clinical Medicine, Research Unit in Periodontology and Periodontal Medicine, University of Florence, Florence, Italy; 2 Department of Experimental and Clinical Biomedical Sciences, Radiology Emergency Unit, University of Florence, Florence, Italy; 3 Department of Experimental and Clinical Biomedical Sciences, Bone Metabolic Diseases Unit, University of Florence, Florence, Italy

**Keywords:** Cone Beam Computed Tomography, Maxillofacial, Jaws, Osteoporosis, Bone Mineral Density, Dual X-ray Absorptiometry

## Abstract

**Purpose of Review:**

This study aimed to investigate the potential of Cone Beam Computed Tomography (CBCT) parameters to identify primary osteoporosis in jawbones.

**Recent Findings:**

Early diagnosis of osteoporosis is crucial but challenging. Dual X-ray Absorptiometry (DXA) is the gold standard to measure bone density, nevertheless it has limitations including cost, radiation dose, and 2D imaging. CBCT is a volumetric imaging technique commonly used in dentistry that showed promise as an alternative tool due to its high spatial resolution and excellent analysis of bone structures.

**Summary:**

Mandibular and maxillary CBCT indices showed a strong correlation with Bone Mineral Density (BMD) assessed by DXA. Regional indices (Anterior, Molar, and Posterior), quantitative indices (Computed Tomography Mandibular Index, Computed Tomography Index Superior and Inferior), Radiographic Density, Fractal Dimension, and the qualitative index named Computed Tomography Cortical Index seemed appropriate for the detection of primary osteoporosis. Morphometric parameters for trabecular bone did not show differences between osteoporotic and non-osteoporotic patients. Future studies should assess diagnostic accuracy of such CBCT indices and formulate standardized measurement protocols.

## Introduction

Osteoporosis is a systemic bone disorder marked by reduced bone mass and deterioration of bone microarchitecture, leading to increased fragility and high risk of fractures [1]. (Osteoporosis is defined as a systemic skeletal disease characterized by low bone mass and microarchitectural deterioration of bone tissue, with a consequent increase in bone fragility and susceptibility to fracture) It is widely recognized that osteoporosis and the consequent fractures are associated with poor quality of life, a reduced independence and an increased mortality [2].

Osteoporosis affects nearly 6% of the world’s population and its incidence has been rising parallel with the aging of the population [3]. Osteoporosis affects individuals of all ages and races, but the risk is higher among postmenopausal females as age and oestrogens deficiency are both recognized as factors associated with a reduction in bone mass [4, 5]. The problem with osteoporosis is that no sign of the disease is manifested until a fragility fracture occurs, therefore most patients with osteoporosis remain untreated. Timely diagnosis of osteoporosis is of utmost importance as it can significantly improve the quality of life and reduce the financial burden of therapeutic approaches [6]. For this reason, intense interest exists within the medical community in developing accurate early diagnostic techniques [7].

Bone mineral density (BMD) values are usually measured in the femur (femoral neck) and/or lumbar vertebra (L1-L4) via dual-energy-X-ray absorptiometry (DXA). Despite DXA is considered the gold standard for the diagnosis of osteoporosis [8], it has some disadvantages as follows: high costs and X-ray doses that limit its use as screening tool on a vast scale; the accuracy and precision of scanning depending on the machine, operator, and patient-related factors; challenges in aligning the device with the jaws, and long scanning time [9–11]. In addition, DXA is a two-dimensional imaging technique that investigates cortical and trabecular parts of bone superimposed, although, in reality, these two parts of bone have different responses and should be investigated separately [12].

Cone Beam Computed Tomography (CBCT) is a volumetric imaging technique commonly used for dental purposes. Recent research found a strong correlation between DXA and CBCT, suggesting that CBCT examinations could be a useful tool to assess BMD and fracture risk, thereby supporting more effective disease monitoring and enhancing clinical management [13]. Moreover, CBCT is widely accessible, offers short scanning times, produces low metal artifacts, provides high image quality for bone structures, and enables volumetric bone analysis [11, 13, 14].

The aim of the study was to review systematically the literature on the potential of CBCT parameters in the identification of primary osteoporosis in jawbones.

## Materials and Methods

### PICO Question

The question was framed using the PICO method. PICO is an acronym that allowed to identify the four elements that are essential to formulate the clinical question and got a targeted answer for the research purpose.

In this investigation the authors questioned whether it was possible to detect structural changes in mandibular and maxillary bones (P) by comparing CBCT (I) to DXA as reference standard (C) in order to identify primary osteoporosis (O).

(P) = population: mandibular and maxillary bones.

(I) = intervention: CBCT examinations.

(C) = comparison: DXA.

(O) = outcome: identifying primary osteoporosis.

The literature searches were planned by selecting keywords based on the above-mentioned PICO question.

### Study Selection, Literature Searches and Data Extraction

The research was conducted until the 31 May 2025 on PubMed, Web of Science, Embase, and Scopus databases and it included articles written in English language. No restriction on the year of publication was imposed during the search.

The selected keywords based on PICO question were “CBCT”, “Cone Beam”, “mandib*”, “maxill*”, “jaw”, “jaws”, “osteoporo*”, “Mineral Density”, and “Low Bone Density”.

Search terms were combined using the Boolean operator “OR” to group the terms related to CBCT, that represents the diagnostic investigation tool. The Boolean operator “OR” was also used for the terms indicating the investigated anatomical areas and for those indicating the pathological condition. Eventually, these three groups of terms were combined using the Boolean operator “AND” to obtain a cross-referenced search between the CBCT imaging technique, the jawbones anatomical area, and the osteoporotic population (Table 1).

**Table 1:** Search strategy and search strings for searches on pubmed, web of science, embase, and scopus. *ti* title, *ab* abstract, *ab, ti* abstract/title, *: truncation of search terms

Indexing Terms	Pubmed (*N*)	Web of Science (*N*)	Embase (*N*)	Scopus (*N*)
#01 CBCT [Title]	2,847	5,894	4,294	711
#02 “Cone Beam” [Title]	9,699	15,530	10,761	4,400
#03 Mandib* [Title/Abstract]	115,471	158,403	124,588	318
#04 Maxill* [Title/Abstract]	119,284	169,709	136,177	611
#05 Jaw [Title/Abstract]	38,361	102,348	45,639	172
#06 Jaws [Title/Abstract]	14,454	102,348	14,257	172
#07 Osteoporo* [Title]	41,557	72,562	61,497	7,506
#08 “Mineral Density” [Title]	14,652	26,603	21,688	36,663
#09 “Low Bone Mass” [Title]	431	828	684	6,499
#10 = #01 OR #02	12,206	20,666	14,501	8,504
#11 = #03 OR #04 OR #05 OR #06	236,202	362,027	263,701	67
#12 = #07 OR #08 OR #09	55,259	97,533	81,768	898
#13 = #10 AND #11 AND #12	35	43	35	5
Pubmed	(“Cone Beam“[Title] OR “CBCT“[Title]) AND (“maxill*“[Title/Abstract] OR “mandib*“[Title/Abstract] OR “Jaw“[Title/Abstract] OR “Jaws“[Title/Abstract]) AND (“osteoporo*“[Title] OR “Mineral Density”[Title] OR “Low Bone Mass”[Title])			
Web of Science	(TI=((“Cone Beam” OR CBCT) AND (osteoporo* OR “Mineral Density” OR “Low Bone Mass”))) AND (AB=((Maxill* OR Mandib* OR Jaw OR Jaws)) OR TI=((Mandib* OR Maxill* OR Jaw OR Jaws)))			
Embase	(‘cone beam’:ti OR cbct: ti) AND (maxill*:ab, ti OR mandib*:ab, ti OR jaw: ab, ti OR jaws: ab, ti) AND (osteoporo*:ti OR ‘mineral density’:ti OR ‘low bone mass’:ti)			
Scopus	(‘cone beam’:ti OR cbct: ti) AND (maxill*:ab, ti OR mandib*:ab, ti OR jaw: ab, ti OR jaws: ab, ti) AND (osteoporo*:ti OR ‘mineral density’:ti OR ‘low bone mass’:ti)			

The extracted data from each study were as follows: study author and publication year, number and gender of individuals enrolled, average age, anatomical area, field of view (FOV) and voxel size, CBCT parameters, CBCT reconstruction planes, clinical question, medical condition, and medications affecting bone metabolism.

### Inclusion and Exclusion Criteria

Studies needed to satisfy the following criteria to be included in this systematic review.

*Inclusion criteria*:

- Osteoporotic/Osteopenic patients.
- CBCT as diagnostic investigation tool.
- DXA as reference standard.
- Jawbones of living beings.

*Exclusion criteria*:

- No osteoporotic/Osteopenic patients.
- Secondary osteoporosis.
- CBCT outside the jawbones.
- No DXA as reference standard.
- Studies conducted on animals or in vitro.
- Use of artificial intelligence to process CBCT examinations.
- Systematic reviews, short communications, letters to the editor, and case reports.

### Risk of Bias Assessment

The authors referred to QUADAS-2 (Quality Assessment Tool for Diagnostic Accuracy Studies-2) to assess the risk of bias. The QUADAS-2 considered four domains, specifically patient selection, index test, reference standard, and flow and timing. For each of these parameters it asked to assess the risk of bias and for the first three of them it also asked to assess the applicability of the included studies to the specific research questions of our review. For each domain, the QUADAS-2 tool proposed specific questions to facilitate evaluation. Responses such as “yes,“, “no”, or “unclear” were adopted to classify the risk of bias as “low”, “high”, or “unclear” respectively, in accordance with the QUADAS-2 guidelines.

## Results

### Study Selection and Data Extraction

**Fig. 1:**
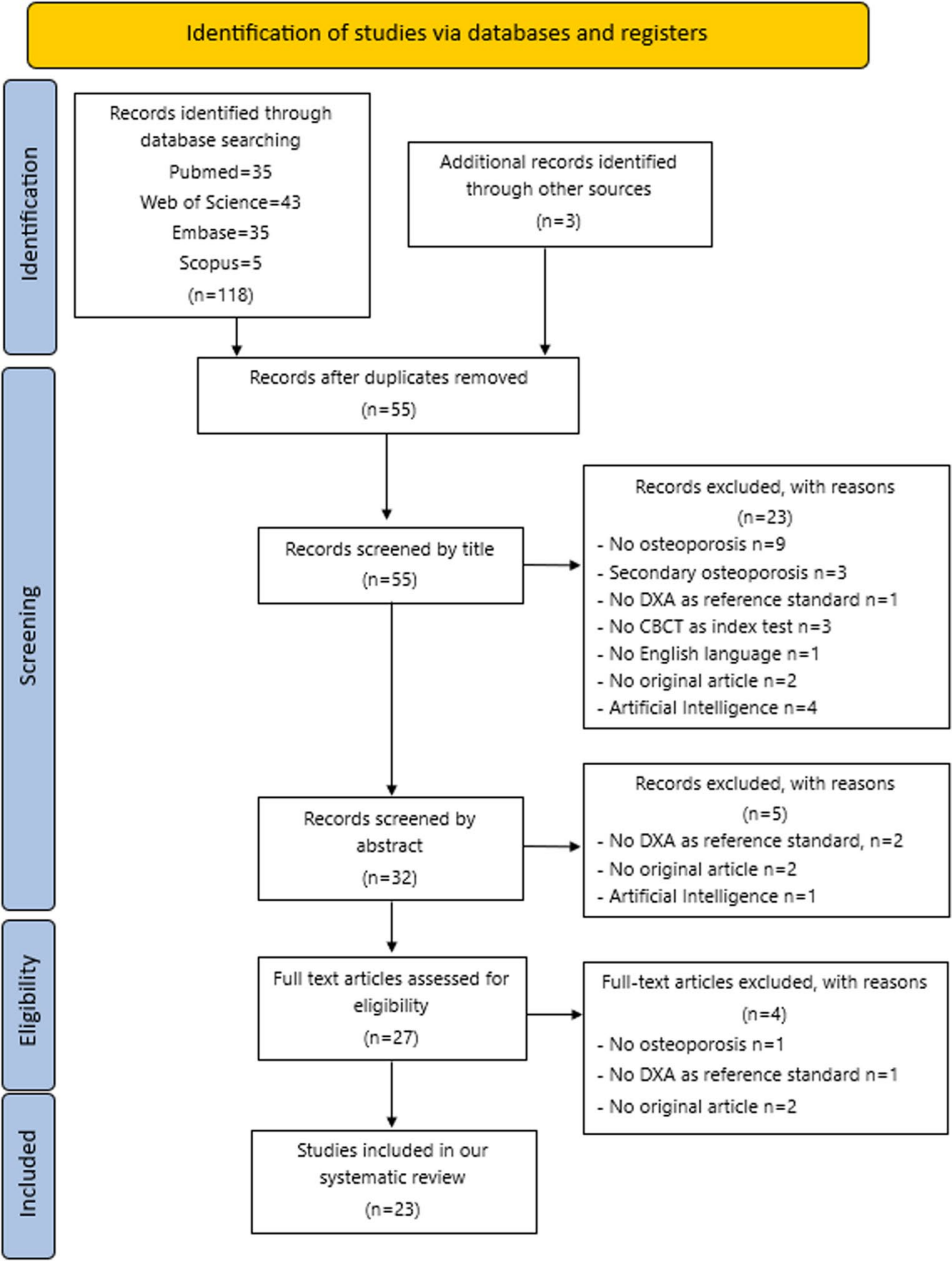
Flow diagram following the PRISMA statement for systematic review. DXA: dual X-ray absorptiometry. CBCT: cone beam computed tomography

The four databases search gave back fifty-two articles after duplicates removing. In addition, three articles were selected through manual research examining the references of the eligible articles. Each article was evaluated for title and abstract and twenty-eight articles were excluded since they did not satisfy the inclusion criteria. A full text review of the twenty-seven selected articles was made and four more articles were excluded. Finally, twenty-three eligible articles were identified out of the one hundred and twenty-one obtained with the research. The flowchart of the paper selection process was shown in Fig. 1.

The twenty-three articles included in the current systematic review were published between 2011 and 2025. Three of them included males and females [11, 15–17], but most of them enrolled only postmenopausal women [6, 12, 18–34]. All the studies investigated mandibles, in addition five of them investigated maxillary bones [11, 15, 16, 20, 31].

Extracted data were shown in Table 2.

**Table 2:** Data extracted from the articles included in the current review. Refer to the Table 3 for the definition of CBCT parameters. *F* female, *M* male, *NA* not available. *: CTMI and MCW are two different ways to name the same parameter. CTCI, MCI and Klemetti Index are three different ways to name the same parameter

Author (year of publication)	*N*° subjects, gender	Mean age (range)	Anatomical area	FOV and Voxel size	CBCT parameter*	CBCT recon-struction plane	Clinical question	Drug	Medical condition
Abdinian M (2023)	80, F	57 (40–77)	Mandible	NA, 125 μm	CTI(I), CTI(S), CTMI	Coronal	Dental treatment	No drug	Normal. Osteopenia, Osteoporosis
Albayati RM (2018)	60, F	Normal: NA (20–30) Osteopenia: NA (> 50) Osteoporosis: NA (> 50)	Mandible	180 × 206 mm, 300 μm	RD, alveolar bone height	Sagittal	Implant planning	No drug	NA
Barngkgei I (2014)	38, F	57 (46–75)	Mandible	130 × 150 mm, 250 μm	RD	Axial	NA	NA	Normal, Osteopenia, Osteoporosis
Barngkgei I (2015)	38, F	57 (47–75)	Mandible and maxilla	130 × 150 mm, 250 μm	Tb.Th, Tb.Sp, BV/TV, BS/TV, trabecular CD	Sagittal, Coronal	NA	NA	Normal, Osteopenia, Osteoporosis
Barra SG (2020)	48, F	61 (51–83)	Mandible	50 × 37 mm, 76 μm	S, A, M, P	Cross	Implant planning	No drug	Normal, Osteopenia, Osteoporosis
Bilgili E (2023)	48, F	53 (NA)	Mandible	160 × 80 mm, 125 μm	Tb.Th, Tb.Sp, BV/TV	Axial	Dental treatment	NA	Normal, Osteoporosis
Brasileiro CB (2017)	18, F	Normal: 56 (47–78) Osteopenia: 58 (50–66) Osteoporosis: 62 (52–80)	Mandible	50 × 37 mm, 76 μm	CTI(I), CTI(S), CTMI	Cross	Implant planning	No drug	Normal, Osteopenia, Osteoporosis
Carvalho BF (2011)	60, F	NA (> 45)	Mandible	200 × 80 mm, 250 μm	FD	Panorex	Implant planning	No drug	Normal, Osteoporosis
De Castro JGK (2020)	103, F	NA (> 45)	Mandible	80 × 80 mm, 250 μm	3D MOI (3D MOI CS (MCW) + 3D MOI PR (MCW) + 3D MOI CQ)	Cross	Dental treatment	NA	Normal, Osteoporosis
Diniz-Freitas M (2014)	46, F	NA (> 45)	Mandible	160 × 60 mm, 300 μm	CTMI, MBMF	Panorex, Cross	Dental treatment	On treatment	Normal, Osteoporosis
Güngör E (2016)	90, M, F	NA (> 30)	Mandible and maxilla	130 × 100 mm, 300 μm	CTI(I), CTI (S), CTMI, RD (CT values), FD, HA	Cross	Implant planning	NA	Normal, Osteopenia, Osteoporosis
Kanneppady SS (2025)	100, NA	NA (45–75)	Mandible	NA	CTMI, RD	NA	NA	NA	Normal, Osteoporosis
Kato CN (2019)	54, F	58 (49–80)	Mandible	50 × 37 mm, 200 μm	CTCI	Panorex	Implant planning	No drug	Normal, Osteopenia along with Osteoporosis
Koh KJ, Kim KA (2011)	21, F	66 (55–72)	Mandible	NA	CTI (I), CTI (S), CTMI, CTCI	Axial, Coronal, Sagittal	NA	NA	Normal, Osteopenia along with Osteoporosis
Mallick SC (2024)	NA, F	NA	Mandible	110 × 80 mm, 75 μm	S, A, M, P	Cross	NA	NA	Normal, Osteopenia, Osteoporosis
Mostafa RA (2016)	50, F	NA (55–70)	Mandible	80 × 80 mm, 200 μm	CTCI, CTMI, CTI	Coronal, Sagittal	Research purpose	No drug	Normal, Osteoporosis
Poiana I (2024)	104, F	NA (> 50)	Mandible	NA	CTI (I), CTI (S), CTMI	Coronal	Implant planning	On treatment and not on treatment	Normal along with Osteopenia, Osteoporosis
Poiana I (2024)	104, F	NA	Mandible	NA	S, A, M, P	Cross	Implant planning	On treatment and not on treatment	Normal along with Osteopenia, Osteoporosis
Sghaireen MG (2020)	81, M(59), F(22)	57 (36–75)	Mandible and maxilla	NA, 200 μm	RD	NA	Implant planning	No drug	Normal, Osteoporosis
Shgaireen MG (2023)	187, M(82), F(105)	NA (45–65)	Mandible and maxilla	NA	RD	NA	Implant planning	NA	Normal, Osteoporosis
Shokri A (2019)	61, F	64 (48–80)	Mandible and maxilla	130 × 140 mm, NA	RD	Cross	Implant planning	NA	Normal, Osteopenia, Osteoporosis
Slaidina A (2023)	127, F	70 (52–91)	Mandible	230 × 115 mm, 300 μm	BV, Tb.BV, BV-Tb.BV	Axial, Coronal, Sagittal	Implant planning	No drug	Normal, Osteopenia, Osteoporosis
Springe B (2014)	45, F	72 (54–85)	Mandible	NA	Height and width of residual ridge	Sagittal	NA	No drug	Normal, Osteopenia, Osteoporosis

### Main Outcomes of the Studies Included in the Review

The papers selected showed high heterogeneity on CBCT parameters and anatomical subsites where these parameters were measured. The current study gathered twenty-six different parameters in twenty-three studies (Table 3).

**Table 3:** Cone beam computed tomography (CBCT) indices in the included studies. *VOI* volume of interest

CBCT Parameter		Description	Unit
3D MOI	Composite CBCT-Driven Osteoporosis Index	A new composite CBCT-driven osteoporosis index (3D MOI) which includes 3 measurements: two quantitative measures evaluating MCW (mandibular cortical width) on panoramic reconstruction images (3D MOI PR) and on cross-sectional images (3D MOI CS), and one qualitative measure assessing cortical bone quality (3D MOI CQ)	NA
A	Anterior Index	The thickness in millimetres of the inferior cortex of the mandible in the cross-sectional image 10 mm anterior to the cross-sectional image through the mental foramen	mm
Alveolar Bone Height	‒	Measure of a line drawn from top to one millimetre just above the inferior alveolar canal in sagittal view	mm
Bone Height	‒	In the midline, measured on a line parallel to the axial axis of the jawbone, which connects the most upper and lower points of the mandible. Mental foramina regions: measured from the lower border of the mandible to the lower border of the mental foramen	mm
Bone Width	‒	Measured on a line perpendicular to height measurements at 2-mm intervals. Measured 10 to 22 mm above the lower mandibular border in the midline, and 8 mm above the lower borders of the mental foramina in the regions of the mental foramina	mm
BS/TV	Bone Surface Density	Ratio of the trabecular bone surface to the total bone volume	mm^2^/mm^3^
BV	Bone Volume	Total volume of bone within the VOI	mm³
BV/TV	Bone Volume Fraction	The portion of mineralized bone tissue in the total volume	%
BV-Tb.BV	Cortical Bone Volume	Total volume of cortical bone within the VOI	mm³
CD	Connectivity density	Represents trabecular connections divided by the total volume	NA
CT value	Computed Tomography value	Quantitative measurement of radio density on a grayscale that refers to the absorption/attenuation coefficient of radiation within a tissue	Hounsfield Units
CTCI = MCI = Klemetti Index	Computed Tomography Cortical Index = Mandibular Cortical Index	The type of the inferior mandibular cortex:	NA
		Type 1: the cortical endosteal margin appears even and regular	
		Type 2: the endosteal margin shows semilunar defects or 1 to 3 layers of cortical endosteal residues	
		Type 3: the cortical layer has numerous (> 3) endosteal residues and is clearly porous	
CTI	Computed Tomography Index	The ratio of the inferior cortical width to the distance between the middle of the mental foramen and the inferior mandibular cortex	NA
CTI (I)	Computed Tomography Index (Inferior)	The ratio of the inferior cortical width to the distance from the inferior margin of the mental foramen to the inferior border of the mandible in coronal or sagittal CBCT images	NA
CTI (S)	Computed Tomography Index (Superior)	The ratio of the inferior cortical width to the distance from the superior margin of the mental foramen to the inferior border of the mandible in coronal or sagittal CBCT images	NA
CTMI = MCW	Computed Tomography Mandibular Index = Mandibular Cortical Width	The inferior cortical width of the mandible in the mental foramen region in coronal CBCT images	mm
FD	Fractal Dimension	Indicator of surface complexity of an object, which quantifies how that object’s surface fills space	NA
HA	Histogram analysis	The brightness and colour information of a specific region on a digital image transformed into a numerical expression	NA
M	Molar Index	The thickness of the inferior cortex of the mandible in the cross-sectional image 10 mm posterior to the cross-sectional image through the mental foramen	mm
MBMF	Mandibular border-mental foramen	Distance from the inferior mandibular border to the inferior margin of the mental foramen	mm
P	Posterior Index	The thickness of the inferior cortex of the mandible in the cross-sectional image 25 mm posterior to the cross-sectional image through the mental foramen	mm
RD	Radiographic Density	Radiographic density in Gray Values	Numerical value
S	Symphysis Index	The thickness of the inferior cortex of the mandible in the cross-sectional image equidistant from the centres of the right and left mental foramina	mm
Tb.BV	Trabecular Bone Volume	Total volume of trabecular bone within the VOI	mm³
Tb.S = Tb.Sp	Trabecular Separation = Trabecular Spacing	Mean distance between the trabeculae	mm
Tb.Th	Trabecular Thickness	Mean thickness of the trabeculae	mm

Quantitative radiomorphometric indices. Computed Tomography Mandibular Index (CTMI), Computed Tomography Index Inferior (CTI (I)), and Computed Tomography Index Superior (CTI (S)) emerged to be significantly correlated with BMD based on DXA at femoral neck and lumbar vertebrae, with lower values in osteoporotic patients than in the control group [6, 15, 28, 30, 35]. Moreover, in one study [22] CTMI, CTI (S), and CTI (I) were significantly lower in osteoporotic patients than osteopenic ones, whereas CTMI and CTI (S) were lower in osteopenic patients than in the control group. On the contrary, one paper 27 did not found any differences in CTMI between osteoporotic and normal BMD groups.

Qualitative index. Computed Tomography Cortical Index (CTCI), also called Mandibular Cortical Index (MCI) or Klemetti Index, showed statistically significant differences between osteoporotic and control groups. In details, the osteoporotic group scored higher number of Type 2 cortical feature, represented by endosteal margin semilunar defects or one to three layers of cortical endosteal residues, followed by Type 3 cortical feature, with major than three endosteal residues in the cortical layer and clear porosity. The control group scored higher number of Type 1 cortical feature corresponding to regular cortical endosteal margins [26–28].

Morphometric parameters for trabecular bone. Trabecular Thickness (Tb.Th), Trabecular Separation (Tb.S or Trabecular Spacing, Tb.Sp), Bone Surface Density (BS/TV), and Connectivity Density (CD) did not show statistically significant differences between osteoporotic and non-osteoporotic women in either of the two studies in which they were analyzed [12, 20]. On the other hand, Bone Volume Fraction (BV/TV) showed statistically significant differences in Bilgili et al. [12], whereas no significant difference was observed in Barngkgei et al. [20].

Regional indices. Three studies evaluated regional indices [21, 29, 34]. Differences between osteoporotic and normal BMD groups were found for Molar and Posterior indices in one study [21, 29, 34] and for Anterior, Molar, and Posterior indices in another one 29. In addition, Anterior, Molar, and Posterior indices showed statistically significant positive correlation with T-score and good positive predicting value for the identification of osteoporosis [29, 36].

Radiographic Density (RD). RD measurements emerged to be significantly lower in osteoporotic patients than in the control group, with higher Gray Scale values and T-values at all anatomical jawbone subsites in the control group [11, 16, 18, 19, 31]. In addition, a significant correlation was found between BMD and RD in different subsites of maxillary and mandibular bones [19, 31, 35]. Particularly, both the trabecular and the trabecular plus cortical bone segments at maxillary tuberosity and also the bone segment at both posterior maxilla and anterior mandible [11] were the areas that best predict the DXA T-score of femoral neck and lumbar spine [11, 31].

Other indices. Total Mandibular Volume and Cortical Bone Volume (BV, BV–Tb.BV) differed significantly between osteoporotic and normal BMD groups, as well as between osteopenic and normal BMD groups, in all anatomical areas except the molar one. These differences were characterized by an increase in trabecular bone volume and a reduction in cortical bone volume, in parallel with an overall decrease in BMD [33].

Height and width of mandibular bone were two indices that did not vary significantly and were not correlated with BMD changes in according to Albayati et al. and Springe et al. [18, 32].

Eventually, Fractal Dimension (FD) was significantly lower in osteoporotic patients than in the normal BMD group [15, 23].

Main outcomes of the studies included in the review were summarized in Table 4.

**Table 4:** Main outcomes of the studies included in the review. Refer to the Table 3 for the definition of CBCT parameters. *DXA* Dual X-ray Absorptiometry, *CBCT* Cone Beam Computed Tomography, *BMD* Body Mass Density, *HU* Hounsfield Unit

	Main outcomes
Abdinian M (2023)	CTMI significantly correlated with vertebral and femoral BMD.Statistically significant correlation between CTI (S) / CTI (I) and vertebral / femoral BMD.CTI significantly lower in osteoporotic group than in control group. Significant positive correlation between CTI and lumbar spine BMD.
Albayati RM (2018)	RD in Gray values significantly higher in control group than in osteoporotic group.
	No statistically significant differences in alveolar bone height between osteoporotic and control groups.
Barngkgei I (2014)	From weak to medium correlation of RD measurements with femoral neck T-scores. Moderate correlation with lumbar T-score for all variables from the ramus and mandibular body, except than one.
	Low sensitivity and high specificity values for such variables.
Barngkgei I (2016)	No statistically significant difference between osteoporotic and non-osteoporotic women for jawbones derived measurements (Tb.Th, Tb.Sp, BV/TV, BS/TV, trabecular CD).
Barra SG (2020)	Statistically significant difference in S index between osteoporotic and osteopenic groups, in M and P indices between osteoporotic and control groups, and in M index between osteopenic and control groups.
	Low sensitivity and high specificity for such indices.
Bilgili E (2023)	Statistically significant difference in BV/TV between osteoporotic and control groups. No difference for Tb.Th and Tb.Sp.
Brasileiro CB (2017)	CTMI lower in osteoporotic group than in normal BMD group, in osteoporotic group than in osteopenic group, and in osteopenic group than in normal BMD group.
	CTI (S) and CTI (I) significantly lower in osteoporotic group than in control group and in osteoporotic group than in osteopenic group, and CTI (S) lower in osteopenic group than in control group.
Carvalho BF (2011)	Mean FD measurements significantly lower in osteoporotic group than in normal BMD group.
De Castro JGK (2020)	Significantly different values of 3D MOI index between osteoporotic and normal BMD groups, with a lower mean value in osteoporotic group than in normal BMD group.
	Association between BMD and three variables, namely 3D MOI (3D MOI CS (MCW)+3D MOI PR (MCW)+3D MOI CQ).
Diniz-Freitas M (2014)	No statistically significant difference for MBMF between osteoporotic and control groups.
	MCW values significantly higher in osteoporotic women on treatment with oral bisphosphonates than in normal BMD group.
	Significant inverse correlation between MCW and the duration of the treatment with oral bisphosphonates.
Güngör E (2016)	CTMI lower in osteoporotic group than in normal BMD group.
	CTMI significantly correlated with vertebral and femoral BMD.
	CTI (S) and CTI (I) significantly lower in osteoporotic group than in normal BMD group.
	Statistically significant correlation between CTI (S) / CTI (I) and vertebral / femoral BMD.
	Mean FD measurements significantly lower in osteoporotic group than in osteopenic and normal BMD groups.
	HA measurements significantly lower in osteoporotic group than in osteopenic and control groups; osteopenic group values were significantly lower than in control group.
	Positive correlation between vertebral BMD and HA measurements in the mandible.
Kanneppady SS (2025)	MCW notably reduced in osteoporotic patients.
	Significant decrease in osteoporotic group for HU values from CBCT measurements.
	Strong positive correlation between HU values and DXA BMD.
Kato (2019)	MCI (or CTCI) obtained by panorex of CBCT with 25 mm slice thickness best identified low-BMD among postmenopausal females.
Koh KJ (2011)	No difference between osteoporotic and normal groups in CTMI.
	CTI (S) and CTI (I) significantly lower in osteoporotic group than in control group.
	Statistically significant difference in CTCI scores between osteoporotic and control groups.
	Osteoporotic group: higher number of Type 2 cortical features followed by Type 3 cortical features. Control group: higher number of Type 1 cortical features.
	Statistically significant correlation between CTCI scores and vertebral BMD.
Mallick SC (2024)	A, M, P indices best predicted osteoporosis with a positive predictive value of 80.8%.
Mostafa RA (2016)	CTMI lower in osteoporotic group than in normal BMD group.
	CTMI significantly correlated with the vertebral and femoral BMD.
	Statistically significant difference in the CTCI scores between osteoporotic and control groups.
	Osteoporotic group: higher number of Type 2 followed by Type 3 cortical features; control group: higher number of Type 1 cortical features. Statistically significant correlation between CTCI scores and vertebral BMD.
Poiana I (2024)	Statistically significant difference between all parameters in the two groups, with higher values for normal BMD group. Moderate positive correlation of all parameters with T-score. Highest correlation for CTMI with femoral neck T-score. Effective predictive power for the presence of osteoporosis.
Poiana I (2024)	Significant higher values of all parameters (A, P, M, S) in normal BMD group than in osteoporotic group.
	Significant moderate correlation between A/P indices and femoral neck/lumbar T-score.
	Stronger correlation for M index. No significant correlation between S index and all BMD measurements.
	A and M indices were predictor of osteoporosis with better prediction power than P index.
Sghaireen MG (2020)	Lower Gray Scale values in osteoporotic group than in control group. Higher T-values associated with higher Gray Scale values at all jaw anatomical subsites.
	Gray Scale values at posterior maxilla were able to predict the presence of osteoporosis. Gray Scale values at posterior maxilla and anterior mandible were able to predict T-value.
Sghaireen MG (2023)	CBCT grayscale values can predict T-score values by DXA.
Shokri A (2019)	Statistically significant difference in RD between control and osteoporotic groups for both trabecular bone and trabecular plus cortical bone segments.
	Significant correlation between femoral neck/lumbar T-scores and RD values at maxillary incisors root ad maxillary tuberosity. Maxillary tuberosity best predicted T-score.
Slaidina A (2023)	No significant difference of Tb.BV between groups in any area.
	Significant difference of BV-Tb.BV and mandibular BV between osteoporotic and normal BMD groups and between osteopenia and normal BMD groups in all anatomical subsites, with an increase in the portion of Tb.BV and a decrease in BV-Tb.BV, with an exclusion for the molar subsite.
Springe B (2014)	No statistically significant relationship between BMD and mandibular residual ridge height measurements nor width measurements.

### Treated and Untreated Osteoporotic Patients

In the only study that compared non-osteoporotic patients with osteoporotic patients on treatment, CTMI, also known as Mandibular Cortical Width (MCW), emerged to be significantly higher in the osteoporotic group [37]. This was different from results obtained comparing non-osteoporotic patients with untreated patients with osteoporosis. In such case, MCW showed low values in osteoporotic patients. Higher MCW values were correlated with higher DXA T-scores [26–28]. Moreover, Diniz-Freitas et al. ^25^ found a significant inverse correlation between MCW and the duration of treatment with oral bisphosphonates [37] The authors attributed this discrepancy to the cross-sectional design of the study and to the lack of pre-treatment MCW measurements. They also hypothesized that MCW increased in participants following treatment but remained low due to extremely low baseline MCW values prior to treatment.

### Risk of Bias

The overall risk of bias was medium based on QUADAS-2 tool parameters (Table 5).

**Table 5:** Risk of bias of the included studies (QUADAS-2). ☺ϑ Low Risk. ☹Λ High Risk. ? Unclear Risk

AUTHOR (PUBLICATION YEAR)	RISK OF BIAS				APPLICABILITY CONCERNS		
	PATIENT SELECTION	INDEX TEST	REFERENCE STANDARD	FLOW AND TIMING	PATIENT SELECTION	INDEX TEST	REFERENCE STANDARD
Abdinian M (2023)	☺	?	?	☺	☺	☺	☺
Albayati RM (2018)	☺	?	?	☺	☹	☺	☺
Barngkgei I (2014)	☺	?	☺	?	☹	☺	☺
Barngkgei I (2016)	☺	?	?	☺	☹	☺	☺
Barra SG (2020)	☺	☺	☺	☺	☺	☺	☺
Bilgili E (2023)	☺	?	?	?	☹	☺	☺
Brasileiro CB (2017)	☺	?	?	☺	☺	☺	☺
Carvalho BF (2022)	☺	?	?	☺	☺	☺	☺
De Castro JGK (2020)	☺	☺	☺	☺	☺	☺	☺
Diniz-Freitas M (2014)	☺	?	?	?	☺	☺	?
Güngör E (2016)	?	?	?	☺	☹	☺	☺
Kanneppady SS, (2025)	?	☹	☺	?	?	☺	☺
Kato CN (2019)	☺	☺	☺	☺	☺	☺	☺
Koh KJ, Kim KA (2011)	☺	?	?	☺	☹	☺	☺
Mallick SC (2024)	☺	?	?	?	☹	☺	☺
Mostafa RA (2016)	☺	?	?	☺	☹	☺	☺
Poiana I (2024)	☺	?	?	?	☹	?	☹
Poiana I (2024)	☺	?	?	☺	☹	?	☹
Sghaireen MG (2020)	☺	☺	☺	?	☹	☺	☺
Sghaireen MG (2023)	☺	☺	☺	?	☺	☺	☺
Shokri A (2019)	☺	?	?	?	☺	?	☺
Slaidina A (2023)	☺	?	?	☺	☺	☺	☺
Springe B (2014)	☺	?	?	☺	☹	?	☺

**Fig. 2:**
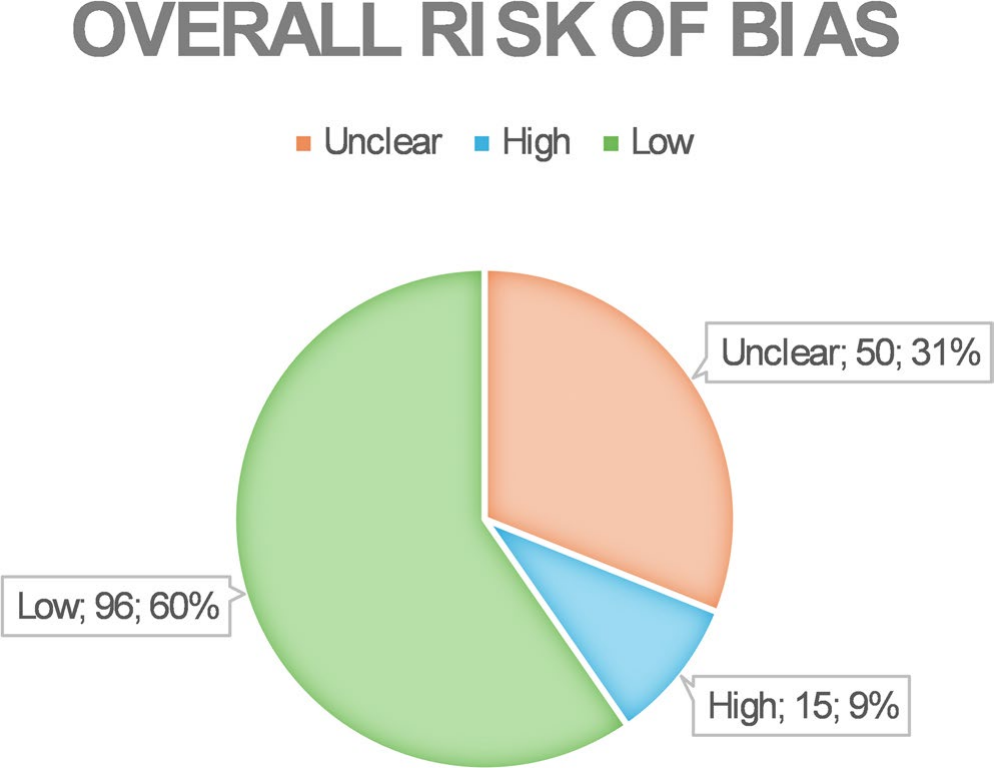
Pie chart depicting the overall percentages of low, unclear, and high risk of bias. Labels reporting showed risk; number of domains; percentage of domains

**Fig. 3:**
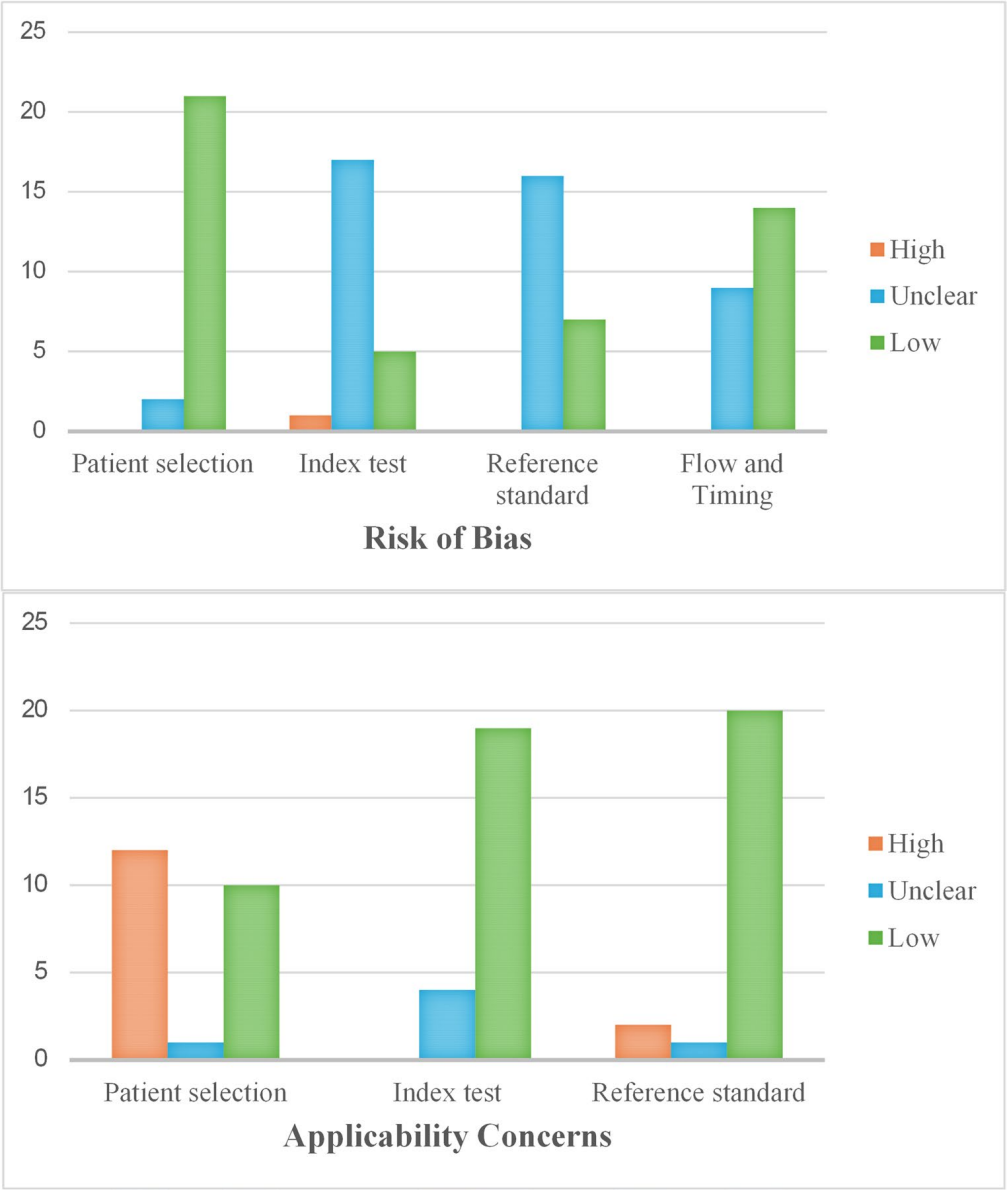
Bar charts illustrating the distribution of risk of bias and applicability concerns within the QUADAS-2 domains

“High risk” of bias was mostly found in the applicability concerns domain for patient selection, since ten studies did not specify if selected patients have or have not taken medications that can affect bone metabolism [11, 12, 15, 19, 20, 23, 27, 31, 34, 35] and three studies included treated and untreated patients in the same sample without a clear partition [29, 30, 38] (Figs. 2 and 3).

Furthermore, “unclear risk” was found in nearly all the studies within the risk of bias domain of index test and reference standard. That was because of the absence of a specified blinding design, so that the operator could be influenced in measuring the index test already knowing the result of the reference standard examination [6, 12, 15, 18–20, 22, 23, 25, 27–34].

Finally, nine articles were assessed as “unclear risk” due to the absence of a clear flow and timing in designing the study [11, 12, 16, 19, 25, 29, 31, 34, 35]. A long timelapse between the acquisition of the reference standard examination and the execution of the index test could preclude a correct comparison between the two tests.

## Discussion

CBCT has been rising popularity in maxillofacial imaging in the last two decades due to its high accessibility, relatively low radiation dose, low metal artifacts, and high spatial resolution [39]. However, the use of CBCT in the evaluation of bone mass and osteoporosis is currently still unstandardized and no universally accepted protocol exists, owing to the high heterogeneity emerged from the investigated studies in the present review on index tests, anatomical areas, and selected population. Twenty-six different indices were gathered in only twenty-three studies and the same index was often measured at different anatomical subsites. Moreover, nineteen out of twenty-three studies enrolled only women subjects and did not consider men. Nevertheless, it is well known that osteoporosis has an overwhelming prevalence in women [4, 5], therefore the sample available to us may be considered representative of the general population.

The current review provided a comprehensive and detailed overview of the indices that may be used to assess the features of osteoporosis in jawbones and their correlation with BMD assessed by DEXA, which represented the reference standard examination. In particular, the most frequently features included quantitative radiomorphometric indices (CTMI, CTI(I), CTI(S)), the qualitative index named CTCI, regional indices (A, M, S, P), RD, and FD. Changes in these indices were strongly correlated with variations in T-score and proved to be considerable tools in the detection of osteoporosis [6, 11, 15, 16, 18, 19, 21–23, 25–31, 34, 35]. As regards radiomorphometric parameters assessing trabecular bone structure (Tb.Th, Tb.S, BS.TV, CD, BV/TV), the current evidence was limited and somewhat contradictory [12, 20, 33].

Despite the substantial number of studies included in this review, it was not possible to determine the diagnostic accuracy of the various parameters since only eight papers carried out diagnostic accuracy studies on overall six indices and an each individual parameter was evaluated in no more than two different studies [19, 21, 23, 24, 26, 30, 31, 34].

The current study assumed that osteoporotic changes in mandibular and maxillary bones were correlated to BMD changes in vertebral or femoral sites, where DXA is normally applied. In fact, the index test ‒ Cone Beam CT ‒ and the reference standard test ‒ DXA ‒ investigated different anatomical areas. The relationship between systemic osteoporosis and jawbone osteoporosis has been extensively debating in the last few years, with numerous studies emphasizing challenges in the establishment of clear and evident results, including the common cross-sectional design of the studies available in literature [40] and difficulties in the application of the same method to evaluate different jawbone subsites [41]. Moreover, maxillary and mandibular bones have unique embryology and physiology. Indeed, the distinction between basal and alveolar bone, the origin of alveolar bone from dental follicle and neural crest cells, as well as the presence of continuous masticatory forces combined with dental movement, are typical features that may account for differences in bone metabolic responses [40, 42]. Nevertheless, the studies included in the current review that showed statistically significant differences between osteoporotic and normal BMD groups, also proved a high correlation between BMD measured by DXA and CBCT measurements [6, 15, 19, 21, 24, 26–28, 31, 35]. Additionally, a recent study by Duncea et al. [43] revealed a moderate correlation between mandibular BMD and lumbar spine, femoral neck, and total hip BMDs, with a considerable reduction in mandibular BMD in women with osteoporosis when compared to those without osteoporosis.

Another aspect worth considering was that most of the studies performed measurements at cortical bone, while it is commonly reported that trabecular bone is extensively affected by hormonal and metabolic changes in osteoporosis [31, 40] and that the earliest signs of structural bone changes are trabecular thinning and loss of connectivity [44]. The few studies in the current review that performed measurements on the upper jaws concluded that maxillary tuberosity, posterior maxilla, and the trabecular part of the bone at the site of mandibular incisors were the areas in which large differences between osteoporotic and control groups were observed [11, 18, 31]. Indeed, maxillary bone is known to have thinner cortical plates and bigger amounts of trabecular bone than mandible. However, changes in cortical bone such as thinning and increasing of porosity may underline osteoporotic changes in mandibular bone, even though they tend to appear later in the disease process [6, 15, 22, 24, 26, 28].

Eventually, we attempted to evaluate CBCT imaging differences among treated and untreated patients with osteoporosis. Unfortunately, only one study focused on bone changes in patients treated for osteoporosis [25]. Therefore, the significance of this comparison was limited. Nevertheless, Diniz-Freitas et al. [25] found significantly higher values of MCW in osteoporotic patients than in the control group and a significant inverse correlation between MCW and the duration of treatment with oral bisphosphonates. Lack of papers on this matter may be explained by a limited interest in the assessment of jawbone osteoporosis in treated patients via CBCT, since such patients already had a confirmed diagnosis of systemic osteoporosis and therefore did not require additional screening with a diagnostic tool different from DXA, unlike the general population or people with undiagnosed osteoporosis.

Two main aspects could have influenced our results introducing risks of bias. The first aspect was that ten out of the twenty-three studies included did not mention if the selected population took medications that can affect bone metabolism and consequently both the bone architecture and the results of the index test. Recent studies suggested that the use of bisphosphonates causes cortical bone structural changes, such as an increase in cortical bone thickness [25, 45, 46]. Thus, the result of the index test ‒ CBCT examination ‒ might not reflect the actual patients’ medical condition, particularly in cases with a large interval between the execution of the reference standard examination and the index test. Secondly, eight studies did not include osteopenic patients, two studies included osteopenic patients in the control group, and two studies considered osteopenia along with osteoporosis. Such heterogeneity in patient group allocation, especially the inclusion of osteopenic patients within the osteoporotic group, might have adversely affected the accuracy of the comparison between the index test and the reference standard. Finally, it is important to note the absence in almost all of the studies of a specified blinded study design. This could have introduced a detection bias since the operator could be influenced in measuring CBCT parameters already knowing the result of the reference standard examination.

Future studies following a strict methodological approach that will focus on evaluating the diagnostic accuracy of various indices could contribute to the development of reliable and standardized diagnostic protocols. Such protocols might also enable the use of CBCT as means of early screening in populations at risk for osteoporosis, giving dentists a significant role in identifying potential osteoporotic patients during their diagnostic workup. This might also reduce the need of additional imaging, specifically DXA, taking into account that CBCT imaging is commonly performed for implant planning and dental treatments, although CBCT cannot currently replace DXA or be considered a primary screening tool.

Furthermore, since dental practices routinely generate CBCT data, this technology can be also implemented with AI for turning already-acquired jawbone CBCT examinations into an opportunistic osteoporosis detection [47, 48]. AI can identify and segment specific anatomical areas such as maxillary/mandibular trabecular bone, mandibular inferior cortex, and mental foramen region. Then, jawbone features including trabecular texture/sparsity, cortical width/thickness/erosion/porosity and radiomorphometric indices can be extracted [49]. The process might continue by estimating whether such extracted osteoporosis-related imaging markers are consistent with low bone mineral density in order to refer high-risk patients for proper osteoporosis workup via DXA [48]. Therefore, AI has been showing a promising tool in recent years as it is fast, eliminates human error and variability among different operators and enables automatic measurement, thereby reducing reporting times. Nevertheless, there are some factors that still limit its clinical applicability, especially the small size of training datasets, the lack of clinical integration and external validation across clinics and CBCT vendors, and the lack of imaging standardization primarily related to the lesser uniformity of CBCT gray values than medical CT Hounsfield units [47–49].

## Conclusions

This systematic review showed a correlation between BMD measured by DXA at femoral neck/lumbar vertebrae and CBCT parameters measurements at maxillary/mandibular bones.

Regional indices (A, M, and P), quantitative indices (CTMI, CTI (S), CTI (I)), RD, FD, and the CTCI qualitative index measured on CBCT imaging proved effective in identifying primary osteoporosis in jawbones, although CBCT cannot currently replace DXA or be considered a primary screening tool. The papers selected in our review were promising in terms of the potential of CBCT, but most of them were still in the experimental phase rather than representing a fully established screening protocol. Future studies could be useful to assess diagnostic accuracy of such CBCT indices and formulate standardized measurement protocols. Finally, this review emphasized the role of dentists in screening possible osteoporotic patients via CBCT during their routinary analysis of dental disorders and surgical planning.

## Key References

- Francisco, I.; Nunes, C.; Pereira, F.; Travassos, R.; Ribeiro, M.P.; Marques, F.; McEvoy, M.; Santos, M.; Oliveira, C.; Marto, C.M.; et al. Bone Mineral Density through DEXA and CBCT: A Systematic Review with Meta-Analysis. Appl. Sci. 2023, 13, 5962. 10.3390/app13105962
  - ○ Authors reported a strong correlation between DEXA and CBCT values measured at various anatomical sites and supported the use of opportunistic CBCT scans as a potential tool for assessing bone mineral density and evaluating fracture risk.
- Mistretta, F. et al. A systematic review and meta-analysis on the concept of bone quality in dento-maxillofacial Cone Beam Computed Tomography. Radiol. Med. 2025, 130, 1193-1206. 10.1007/s11547-025-02052-5
  - ○ The study evaluated the role of CBCT in assessing bone quality of mandibular and maxillary bones, and concluded that CBCT, thanks to its high resolution with small voxel size, is considered a suitable tool for the assessment of bone microarchitecture.
- Duncea, I.; Bacali, C.; Buduru, S.; Scrobota, I.; Almășan, O. The Association of Systemic and Mandibular Bone Mineral Density in Postmenopausal Females with Osteoporosis. Medicina 2024, 60, 1313. 10.3390/medicina60081313
  - ○ The study assessed the association between systemic BMD and mandible BMD, performing DEXA scans at both conventional and mandibular sites, and concluded that oteoporosis also affects mandibular bone density.

## Data Availability

No datasets were generated or analysed during the current study.
